# Generalisation to novel exemplars of learned shape categories based on visual and auditory spatial cues does not benefit from multisensory information

**DOI:** 10.3758/s13423-024-02548-7

**Published:** 2024-08-05

**Authors:** A. O’Dowd, R. J. Hirst, M. A. Seveso, E. M. McKenna, F. N. Newell

**Affiliations:** 1 School of Psychology and Institute of Neuroscience, Trinity College Dublin, Dublin, Ireland; 2 Department of Psychology, New York University Abu Dhabi, Abu Dhabi, United Arab Emirates

**Keywords:** Object categories, Shape perception, Multisensory, Audio-visual

## Abstract

Although the integration of information across multiple senses can enhance object representations in memory, how multisensory information affects the formation of categories is uncertain. In particular, it is unclear to what extent categories formed from multisensory information benefit object recognition over unisensory inputs. Two experiments investigated the categorisation of novel auditory and visual objects, with categories defined by spatial similarity, and tested generalisation to novel exemplars. Participants learned to categorise exemplars based on visual-only (geometric shape), auditory-only (spatially defined soundscape) or audio-visual spatial cues. Categorisation to learned as well as novel exemplars was then tested under the same sensory learning conditions. For all learning modalities, categorisation generalised to novel exemplars. However, there was no evidence of enhanced categorisation performance for learned multisensory exemplars. At best, bimodal performance approximated that of the most accurate unimodal condition, although this was observed only for a subset of exemplars within a category. These findings provide insight into the perceptual processes involved in the formation of categories and have relevance for understanding the sensory nature of object representations underpinning these categories.

**Supplementary Information:**

The online version contains supplementary material available at 10.3758/s13423-024-02548-7.

## Introduction

The capacity to organise stimuli into discrete categories underpins the ability to identify, recognise and interact with the external world (Goldstone & Hendrickson, 2010; Pérez-Gay et al., 2017). Often, category knowledge is acquired (Pérez-Gay et al., 2017) via the combination of ‘bottom-up’ and ‘top-down’ mechanisms, and the capacity to organise information into distinct categories has been demonstrated for a range of stimuli (e.g., Folstein et al., 2015; Gaißert et al., 2012; Levin & Beale, 2000; Maddox et al., 2002; Newell & Bülthoff, 2002). An object may be assigned to a particular category based on a combination of its sensory features (e.g., shape, texture, sound; e.g., if categorising based on shape, a lacrosse ball will be categorised with a tennis ball, not a rugby ball), prior knowledge (e.g., semantic) stored in long-term memory (e.g., if categorising based on contact versus non-contact sports, a lacrosse ball will be categorised with a rugby ball, not a tennis ball), and learned expectations of category structures and distributions (e.g., Flannagan et al., 1986).

Despite the multisensory nature of everyday perceptual experiences, the multisensory properties of the brain (Ghazanfar & Schroeder, 2006) and evidence that categories are formed within multiple sensory modalities (e.g., Gaißert et al., 2012; Maddox et al., 2002; Newell & Bülthoff, 2002), it remains unclear how multisensory features or cues influence category formation and representation (Newell et al., 2023). Nevertheless, integrating cross-modal information during category learning (Li et al., 2020a; Maddox et al., 2006; Smith et al., 2014) and transferring category knowledge across modalities (Sun et al., 2023; Yildirim & Jacobs, 2015) is possible. Multisensory object learning during categorisation can benefit perception through feature differentiation in primary sensory cortices and multisensory integration in multiple brain regions (Viganò et al., 2021). Moreover, the representations of familiar objects can be fundamentally multisensory (Matusz et al., 2017; Yildirim & Jacobs, 2012), and object identification and recognition are influenced by exposure to multisensory information (Chen & Spence, 2010; Lehmann & Murray, 2005; Matusz et al., 2017; Molholm et al., 2004; Thelen et al., 2015), likely benefitting the learning process (Shams & Seitz, 2008). Therefore, learning to form object categories based on multiple sensory modalities should facilitate enriched memory representations that enhance recognition (Ernst & Bülthoff, 2004). Using more than one sensory cue to categorise an object may also be an optimal approach to minimise category uncertainty, particularly when category membership is difficult to discern unimodally, as more information is considered. Categorising a ball as a tennis ball or a lacrosse ball based purely on shape would be difficult visually. Therefore, the use of additional auditory cues (e.g., the sound the ball makes when dropped) may help to disambiguate correct category membership. However, categorising a ball as a tennis ball or a rugby ball would be highly efficient based on visual cues alone due to the distinctiveness of these shapes.

This study examined whether object categorisation is improved if category information is acquired from both vision and audition. Participants learned to categorise unfamiliar stimuli that varied in spatial information, with category membership based on shape similarity (Li et al., 2020b). Shape informs visual object recognition (Biederman, 1987; Tarr & Bülthoff, 1998) and similarity (Edelman, 1995; Newell, 1998), and is amenable to categorization effects (e.g., Medin et al., 1993; Newell & Bülthoff, 2002). Recent research suggests that object shape can be perceived using sound information in both sighted (Brown et al., 2011; Haigh et al., 2013; Hertz & Amedi, 2015; Kim & Zatorre, 2008; Proulx et al., 2014) and blind (e.g., Striem-Amit et al., 2012; Ward & Meijer, 2010) individuals, even after a brief learning period. Indeed, shape-based sounds activate regions of the brain that are typically involved in the visual processing of object shapes, such as the lateral occipital cortex (LOC; Amedi et al., 2007; James et al., 2011). These activation patterns are dependent on shape-based processing (Amedi et al., 2007; James et al., 2011): for example, Amedi et al. (2007) reported that activity in the LOC in response to soundscapes was observed only in participants who were trained to interpret shape information from these soundscapes. In the current experiments, the categorisation of novel objects was based on visual-only, auditory-only or audio-visual shape cues. Categorisation was assessed for both learned and novel exemplars to investigate if generalisation to novel objects occurred and whether bimodal cues enhanced such generalisation. It was hypothesised that if multisensory category learning benefits the formation of categories, categorisation performance should be strongest (i.e., higher categorisation accuracy and better generalisation) after bimodal relative to unimodal learning.

## Experiment 1

### Methods

#### Participants

An a priori simulated power analysis was performed using the ‘simr’ package (Green & MacLeod, 2016) in R via Rstudio (Team R, 2021). Based on pilot data (*N* = 21) and 1,000 stimulations, a sample of 70 participants was selected to provide at least 85% power to detect the effects of interest. This was based on likelihood ratio tests which compared model fits with and without these effects (see *Analysis* below) using an adjusted alpha level of *p* = 0.0166667 (considering a maximum of three tests would be applied to each dependent variable across main and interaction effects of interest, the alpha level was adjusted with the Bonferroni correction for multiple comparisons). A slightly more conservative power threshold (≥ 85%) was selected given uncertainty in the expected size of the final effects relative to the pilot data.

In total, 89 individuals participated in the experiment, 87 of whom were recruited using Prolific (https://www.prolific.co/). The two remaining participants were recruited in-person from a convenience sample in Trinity College Dublin (all participants completed the experiment online). All participants were reimbursed at a rate of €11.00 per hour. Inclusion criteria included having no hearing impairments, normal or corrected-to-normal vision, and English as a spoken language, and recruitment was controlled to obtain a balanced sample of males and females. Data were omitted from one participant due to technical issues, 12 participants who did not complete the experiment, and one participant who completed the experiment but scored at or below chance level (50%) in all three modality conditions. The final analysis sample consisted of 75 participants with an additional five participants recruited to correct for imbalanced experimental conditions following randomisation. Descriptive statistics are shown in Table 1.

**Table 1:** Descriptive statistics for the final analysis sample in Experiment 1 (*N* = 75)

Mean (SD) age, years	Sex	No. (%)	Education	No. (%)
34.31 (10.42)	Male	37 (49)	Primary/none	1
	Female	38 (51)	Secondary	13 (17)
			Tertiary	61 (81)
Vision problem^a^	No. (%)	Hearing problem	No. (%)	
Yes	2 (3)	Yes		
No	73 (97)	No	75 (100)	

^a^All participants reporting a vision problem performed above chance level in the visual condition

The study was approved by the Trinity College Dublin, School of Psychology Research Ethics Committee and complied with relevant data protection legislation.

#### Stimuli and materials

**Fig. 1:**
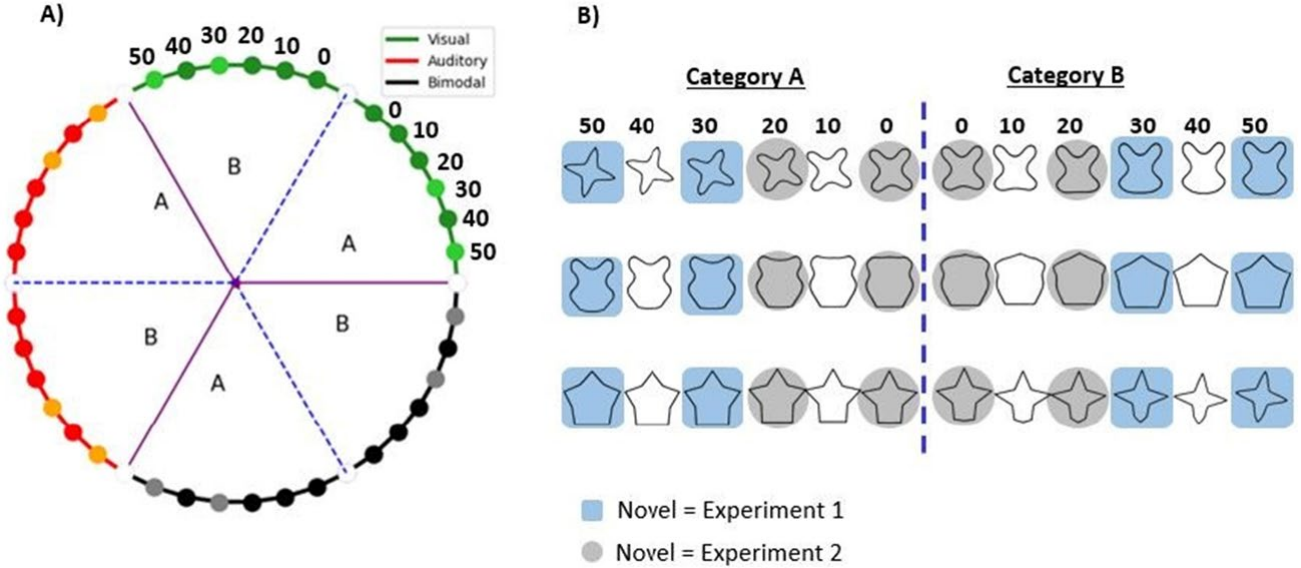
A schematic illustration of the circular shape space representing the different categories of shapes used in Experiments 1 and 2 (adapted from Li et al., 2020b). (**A**) This space is divided into thirds, each assigned to one modality condition (vision, audition, bimodal; an example of one possible configuration is shown). Within each third, 12 positions represented exemplar members of two different categories, with category boundaries demarcated by dashed blue lines: six adjacent exemplars were assigned to either category (‘A’ and ‘B’). The distance from each exemplar to the category boundary is indicated in degrees, such that 0° is next to the category boundary and 50° is the category edge (as demonstrated here for the visual condition). Exemplars included in both category learning and test in Experiment 1 are represented by darker colours (i.e., red, dark green and black) whereas exemplars included in the categorisation test only (in Experiment 1) are represented by lighter colours. (**B**) An illustration of the exemplar shapes used in Experiments 1 and 2 (Li et al., 2020b). The novel exemplars used for category testing are highlighted in blue squares (Experiment 1) and grey circles (Experiment 2). The dashed blue line indicates the category boundary. Each set of 12 exemplars was presented in one of three modality conditions

Visual exemplars were extracted from a visual circular shape space created by Li et al. (2020b). This circular shape space presents 360 two-dimensional shapes, each 1° apart on the circle with the circular space defined by stimulus similarity (i.e., where angularity is a proxy for similarity; Li et al., 2020b). This circular shape space was selected due to its previous validation (Li et al., 2020b), the large stimulus set size, and the inclusion of simple, geometric, novel shapes (without semantic meaning), each equally spaced apart, that could be converted into distinct sound representations (see below). Moreover, previous category studies concerning shape have similarly availed of a continuum of objects to investigate category learning and categorization ability (e.g., Folstein et al., 2015; Gillebert et al., 2009; Newell & Bülthoff, 2002; Panis et al., 2008). These stimulus sets are advantageous in that their stimuli vary in a controlled and gradated manner, representative of the gradual (as opposed to abrupt) variations in object features that often occur naturally in the real world. As the main experiment involved three modality conditions (vision, audition and bimodal), the circle space was divided into thirds, one per modality condition per participant, to prevent any overlap in the exemplars presented across conditions. The allocation of each third of the circular shape space to a modality condition was counterbalanced across participants. Of the 120 possible shapes in each third (see Fig. 1), a subset of 12 shapes was selected with each shape positioned 10° apart on the circle for each modality condition. Of these 12 shapes, six were classified into category ‘A’ and the remaining six into category ‘B’, thus ensuring balanced categories. Of the six sampled shapes per category, two shapes were retained as ‘novel’ items, to be presented at test but not during learning. Hereafter, the positions of the exemplars in a category are defined based on their degrees of separation from the category boundary (0°, 10°, 20°, 30°, 40°, 50°), where 0° is next to the midpoint of each third of the circular shape space, representing the position on the category boundary and 50° is the category edge. The two novel shapes were located at positions 30° and 50° from the category boundary. See Fig. 1 for details.

As in previous audio-visual studies (Amedi et al., 2007; Haigh et al., 2013; Hertz & Amedi, 2015; Kim & Zatorre, 2008; Striem-Amit et al., 2012), the auditory exemplars consisted of soundscapes. Here, the soundscapes represented the geometric shapes of the circular shape space. The soundscapes were created using the Image-to-Audio, Spectrogram Player (2023, https://nsspot.herokuapp.com/imagetoaudio/) with the following settings: 3-s duration, 10,000 Hz and a density setting of 1. The visual shapes were first edited using GIMP (version 2.10.32), in which a Gaussian blur (strength settings: x = 1, y = 1) and colour inversion were applied to facilitate conversion to soundscapes. To verify that each soundscape matched the corresponding visual shape, each soundscape was back transformed into visual shapes using Sonic Visualiser (2023, version 4.5.1). Example soundscapes are available on the Open Science Framework page for Experiment 1 at: https://osf.io/ujyrw/?view_only=35ca39e3c49f49558274dc0dc4bd39f9. The use of these soundscapes as opposed to arbitrary sounds allowed for the presentation of auditory information that was uniquely informative of the specific spatial geometry of the corresponding visual shape.

Bimodal stimuli were created by presenting the visual shape at the same time as the soundscape. The exposure time of the visual-only and auditory-only exemplars was matched by dynamically revealing the visual shape (using an opaque window which moved from left to right) synchronous with the sound of the shape feature. This process had the effect of revealing the visual shape in time with the soundscape. This allowed for a synchronous pairing of the visual and auditory spatial cues in the bimodal condition. This gradual reveal of the visual exemplar was also maintained in the unimodal visual condition to allow for comparison across conditions.

#### Procedure

The experiment was built in PsychoPy (Peirce et al., 2019, version 2022.2.5) and conducted online using Pavlovia (https://pavlovia.org/) on desktops only. The participants were first presented with a study information sheet and asked to complete a consent form to proceed. They were subsequently asked to provide basic demographic information and technical details (see Table 1). The participants were asked to sit in a quiet space and fixate on a fixation cross in the centre of the screen. The experiment began with a simple volume checker, where the participants were instructed to wear head/earphones, increase the volume on their device, and indicate what sound was being played. Failure to indicate the correct sound (white noise) resulted in the termination of the experiment.

**Fig. 2:**
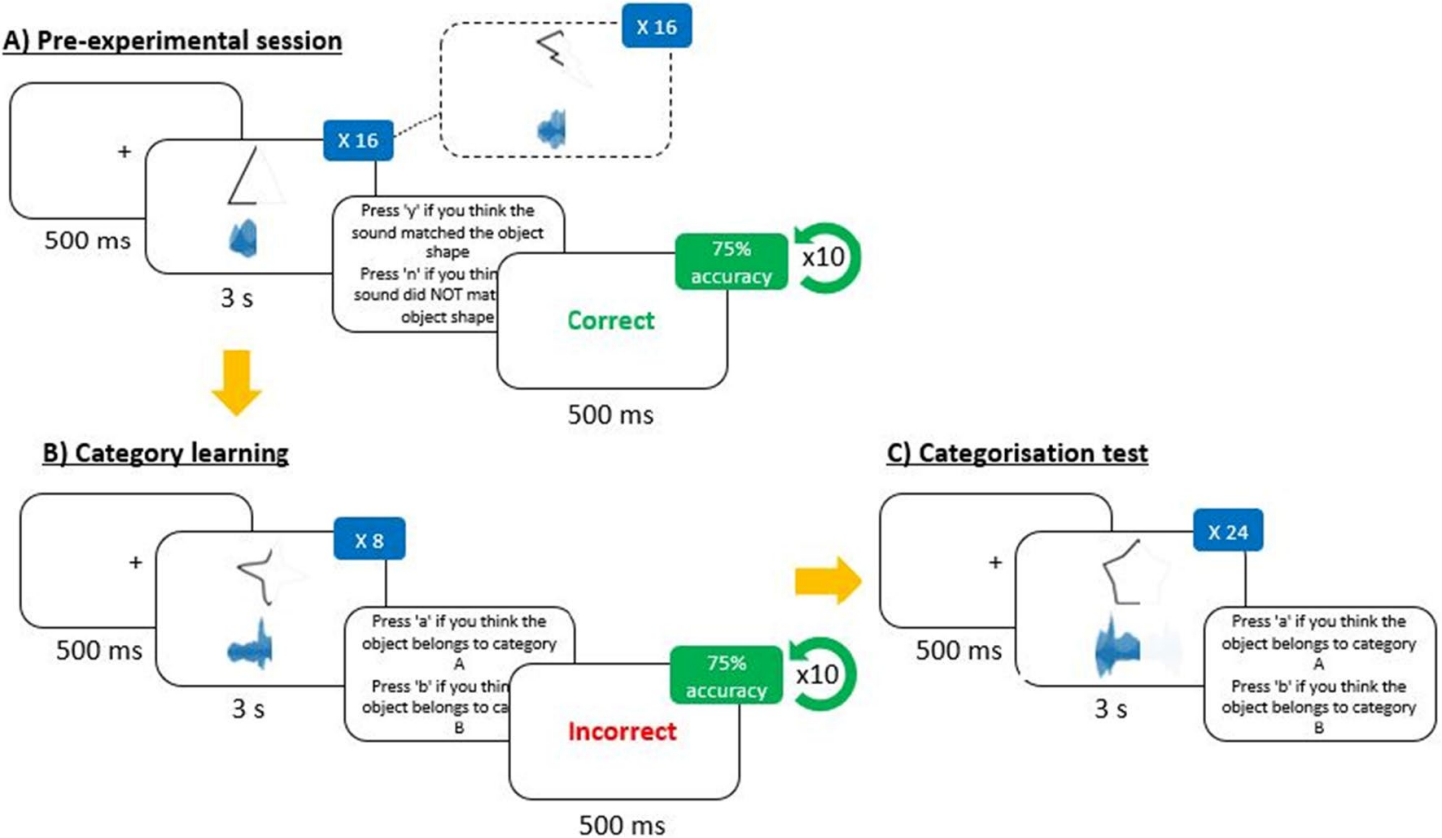
Schematic illustration of the sequence of sessions in Experiment 1. (**A**) Example of a trial sequence from the audio-visual pre-experimental sessions (solid box: simple shape, dashed box: complex shape). Example of a trial in each of the (**B**) category learning phase (bimodal example) and (**C**) categorisation test (bimodal example) in the main experiment. Note that the visual depiction of the sound frequency is for illustrative purposes only and was not shown to the participants in the experiment

To ensure participants were exposed to the spatial correspondence between visual shapes and soundscapes, two pre-experimental sessions were first conducted using stimuli which did not appear in the main experiment (see Fig. 2). In the first session, the participants were presented with eight simple visual shapes (e.g., triangle, circle, flat line) across trials, each with a corresponding soundscape that was either spatially congruent or incongruent (a total of 16 trials, randomly presented). In the second session, more complex shapes (e.g., chevron, lightning bolt, cross) were used, again with a congruent or incongruent sound in each trial. Participants performed a matching task in both sessions in which they indicated whether the visual shapes matched the soundscape (by pressing the ‘Y’ key) or not (by pressing the ‘N’ key). A trial began with a fixation cross (500 ms) followed by the audio-visual stimulus (3 s) then a response prompt, which remained until a response was made. Feedback (‘correct’ or ‘incorrect’, presented for 500 ms) was provided following each response. The participants were informed that 75% accuracy in both sessions was required to proceed to the main part of the experiment. To reach the required 75% accuracy, a maximum of ten repeats of each session was allowed. Otherwise, the experiment was terminated.

The main experiment was based on a within-subjects design, comprising a category learning and subsequent categorisation test which was blocked for each of three learning modalities. In each learning phase, eight different exemplars were presented as visual-only, auditory-only (i.e., soundscapes) or bimodal shapes, and the learning phase was followed directly by a categorisation test in the same modality (i.e., visual learning then visual categorisation, etc.). The order of the modality conditions was counterbalanced across participants and they could take a self-timed break between blocks if necessary. Different sets of eight exemplar shapes were used across learning modalities. Within each modality, four shape exemplars belonged to category ‘A’ (see Fig. 1) and four to category ‘B’. Each exemplar was presented once, resulting in a total of eight randomly ordered trials per learning modality (per repeat of the learning session).

At the beginning of each modality learning phase, participants were informed of the modality condition (visual, auditory or audio-visually) in which the object shapes would be presented and were reminded that the sounds represented the shape of an object and that similar shapes/sounds/shape-sound pairs belonged in the same category. Participants were instructed to press 'A' on the keyboard if they thought the exemplar belonged to category ‘A’ or 'B' if it belonged to category ‘B’. A trial began with a fixation cross (500 ms), followed by the exemplar (3 s) and a response prompt, which remained on-screen until a response was made. Feedback (correct or incorrect presented for 500 ms) was provided following each response. The participants were informed that 75% accuracy was required during the learning phase to proceed to the next part of the experiment. If the required threshold was not reached in a maximum of ten repeats of the learning trials, the experiment terminated.

The categorisation test included the same eight shape exemplars that were just learned plus four novel exemplars, two from category ‘A’ (see Fig. 1) and two from category ‘B’. These novel exemplars were positioned either close to the mid-point of each category or distant to the category boundary (see Fig. 1). Therefore, these novel members were both diagnostic of category membership and varied in their distinctiveness (i.e., distance from category boundary). Each shape exemplar was presented twice across trials, resulting in a total of 24 randomly ordered test trials per modality condition (vision, audition, bimodal). A trial sequence (i.e., fixation, exemplar and response prompt) was the same as in the learning phase. However, no feedback was given during the categorisation test.

In both the category learning and categorisation test, the participants were instructed to respond as quickly and accurately as possible, but no time limit was imposed on responses. Once the main experiment was finished, the participants were debriefed. The experiment took approximately 20 min for each participant to complete.

#### Analysis

The analysis assessed how accurately the participants categorised each exemplar based on all exemplar positions (0°, 10°, 20°, 30°, 40°, 50°, i.e., from closest to most distant from the category boundary) in the categorisation test, and categorisation accuracy was derived for each exemplar position (i.e., in the shape space, see Fig. 1) across categories. Due to practical considerations (length of the online experiment) and the high performance threshold imposed during category learning, there were two trials per exemplar (based on pilot data, this trial number was considered sufficient for detecting differences in performance accuracy across conditions). As such, accuracy took the values of 0 (incorrect categorisation on both trials), 0.5 (correct categorisation on one trial only) or 1 (correct categorisation on both trials). Therefore, a generalised logistic mixed-effects regression model was fitted to these data using the ‘logit’ link. The fixed effects were exemplar position, modality (bimodal, ‘best’ unimodal, ‘worst’ unimodal) and the exemplar position*modality interaction. Unimodal performance was classified into ‘best’ versus ‘worst’ unisensory modality based on each participant’s mean accuracy to assess whether bimodal learning offered a genuine advantage over unimodal learning (e.g., see Scheller & Nardini, 2023). If performance was equivalent across the visual-only and auditory-only conditions (Experiment 1: *n* = 5; Experiment 2: *n* = 5), vision was classified as the best sensory modality in line with the broader pattern exhibited.

The model was adjusted for age, participant sex and category (‘A’, ‘B’), and included a random effect of intercept across participants and a random slope for modality, which was found to improve model fit compared to a model without the random slope term (likelihood ratio test*; p* < 0.001).^1^ The statistical significance of the exemplar position*modality interaction was assessed with a likelihood ratio test, where the fit of the model with and without the interaction term was compared while holding all of the aforementioned terms constant.

The data were analysed using R via RStudio (R Core Team, 2021). Models were fitted using the ‘lme4’ package (Bates et al., 2014). Post hoc comparisons were conducted using the ‘emmeans’ package (Lenth Russell et al., 2022) and Bonferroni correction was applied to correct for multiple comparisons. Experiment 1 was not preregistered, but further details, including the analysis script, are available on the Open Science Framework at: https://osf.io/ujyrw/?view_only=35ca39e3c49f49558274dc0dc4bd39f9.

### Results

For the simple shape and complex shape pre-experimental sessions, overall accuracy (in the final learning block) was 83% (*SD* = 7.6) and 82% (*SD* = 6.5), respectively. To reach at least 75% accuracy for both the simple and complex shapes, an average of 1.9 (*SD* = 1.6) and 2.3 (*SD* = 1.9) learning blocks was required, respectively. For the unimodal visual, auditory and bimodal category learning, overall accuracy (in the final learning block) was 84% (*SD* = 9.1), 80% (*SD* = 7.7) and 84% (*SD* = 9.6), respectively. To reach at least 75% accuracy for unimodal visual, auditory and bimodal category learning, an average of 2.3 (*SD* = 1.5), 3.1 (*SD* = 2.3) and 2.6 (*SD* = 1.4) learning blocks was required, respectively. In the categorisation test, overall accuracy was 82% (*SD* = 39), 68% (*SD* = 47) and 77% (*SD* = 42) for unimodal visual, auditory and bimodal categorisation, respectively. The ‘best’ and ‘worst’ accuracy performance in the unimodal conditions was 88% (*SD* = 32) and 61% (*SD* = 49), respectively. For approximately 75% of the sample (*n* = 56), vision was the ‘best’ modality.

**Fig. 3:**
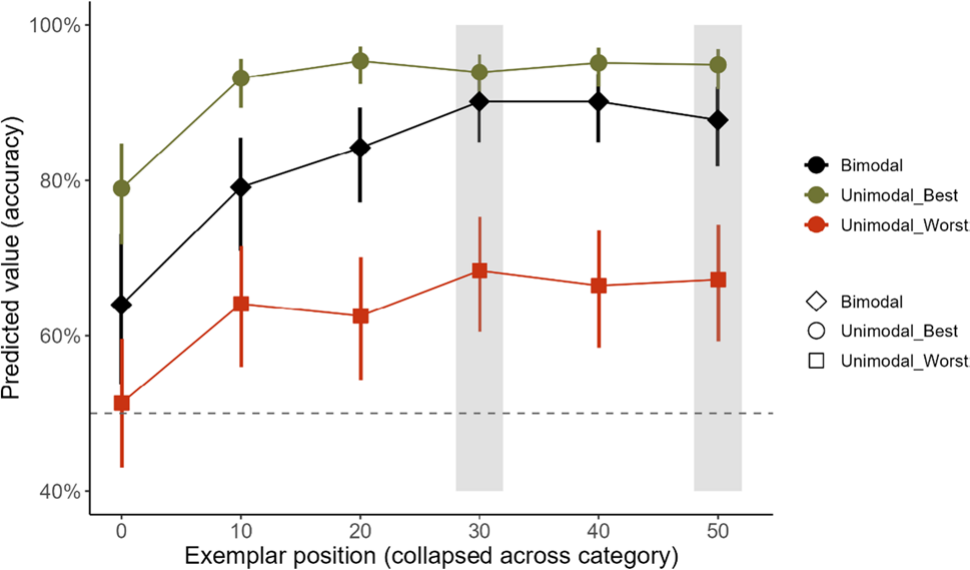
Summary of findings of mixed-effects models predicting categorisation accuracy across exemplar positions relative to the category boundary (see text) and modalities in Experiment 1. Areas shaded grey indicate the position of the novel exemplars. The dashed horizontal line demarcates chance level (50%) performance. Error bars show 95% confidence intervals

The effect of exemplar position (χ^2^(5) = 142.51, *p* < 0.001) and modality (χ^2^(2) = 87.35, *p* < 0.001; see Online Supplementary Materials (OSM)) and the exemplar position*modality interaction (χ^2^(10) = 31.26, *p* < 0.001) significantly contributed to the model predicting categorisation accuracy, as shown in Fig. 3. As expected, exemplars were more accurately categorised in the ‘best’ versus the ‘worst’ unimodal condition. In addition, exemplars at positions 50°, 40°, 30^o^ and 20^o^ on the circular shape space were 3.50 (95% CI [1.48, 8.29]), 4.62 (95% CI [1.92, 11.12]), 4.23 (95% CI [1.75, 10.20]) and 3.19 (95% CI [1.38, 7.39]) times more likely to be accurately categorised in the bimodal versus ‘worst’ unimodal condition, respectively. However, exemplars at positions 20° and 10° were 74% (odds ratio = 0.26, 95% CI [0.09, 0.74]) and 72% (odds ratio = 0.28, 95% CI [0.10, 0.75]) less likely to be accurately categorised in the bimodal versus ‘best’ unimodal condition, respectively. All other contrasts were statistically non-significant at the corrected alpha level. There was no statistically significant difference in categorisation accuracy for any modality for the novel exemplars at positions 50^o^ (‘best’ unimodal: odds ratio = 1.05, 95% CI [0.38, 2.91]; ‘worst’ unimodal; odds ratio = 0.97, 95% CI [0.53, 1.77]; bimodal: odds ratio = 1.27, 95% CI [0.58, 2.80]) and 30° (‘best’ unimodal: odds ratio = 1.27, 95% CI [0.47, 3.41]; ‘worst’ unimodal; odds ratio = 0.92, 95% CI [0.50, 1.68]; bimodal: odds ratio = 1, 95% CI [0.45, 2.24]) versus the exemplar at position 40° (the most similar learned exemplar).

### Discussion

The results of Experiment 1 indicated that participants were able to categorise exemplars defined by similarity of shape and that category learning generalised well to novel exemplars, irrespective of sensory modality and their position relative to the category boundary. However, there was no advantage of bimodal cues for categorisation: categorisation accuracy in the bimodal condition was either comparable to that of the ‘best’ unimodal condition or intermediate to the ‘best’ and ‘worst’ unimodal conditions, depending on the exemplar position within the category. Experiment 2 investigated whether category generalisation would similarly extend across modalities to novel category exemplars positioned closer to the category boundary, thereby being less distinctive or diagnostic of category membership. The rationale for this was that multisensory learning may be more likely to enhance performance in a more challenging categorisation task, based on the principle of inverse effectiveness (Meredith & Stein, 1983). This experiment would allow us to establish whether a benefit of bimodal category information arises when category membership is more difficult to discern and whether bimodal categorisation accuracy for more distinct exemplars is consistently comparable to that of the ‘best’ unimodal condition.

## Experiment 2

### Methods

#### Participants

Given the high degree of similarity in the paradigms featured in Experiment 1 and Experiment 2, the results of the same simulated power analysis described in Experiment 1 were used to inform the sample size of Experiment 2 (*N* = 70). In total, 74 individuals participated in the experiment, 72 of whom were recruited on Prolific (https://www.prolific.co/), using the same inclusion criteria. As in Experiment 1, the two remaining participants were recruited in-person from a convenience sample in Trinity College Dublin (all participants completed the experiment online). The participants who took part in Experiment 1 were excluded from Experiment 2. Data were omitted from three participants who did not complete the experiment and from one participant who completed the experiment but scored at or below chance level (50%) in all three modality test conditions. The final analysis sample consisted of 70 participants. Descriptive statistics are shown in Table 2. All participants provided informed consent before participating in the experiment and were fully debriefed once the experiment was completed.

**Table 2:** Descriptive statistics for the final analysis sample in Experiment 2 (*N* = 70)

Mean (SD) age, years	Sex	No. (%)	Education	No. (%)
36.03 (13.78)	Male	33 (47)	Primary/none	/
	Female	37 (53)	Secondary	8 (11)
			Tertiary	62 (89)
Vision problem^a^	No. (%)	Hearing problem^b^	No. (%)	
Yes	5 (7)	Yes	1	
No	65 (93)	No	69 (99)	

^a^All participants reporting a vision problem performed above chance level in the visual condition

^b^The participant who reported a hearing problem performed above chance level in the auditory condition

#### Exemplars

The same sets of exemplars described in Experiment 1 were used in Experiment 2.

#### Procedure

The same procedure described in Experiment 1 was followed in Experiment 2, featuring a within-subjects design. Once again, the main experiment consisted of category learning and a subsequent categorisation test. However, the four novel exemplars, two from category ‘A’ and the same two from category ‘B’, introduced in the categorisation test were taken from positions that were either directly beside (i.e., position 0°) or closer to (i.e., position 20°) the category boundary compared to the exemplars used in Experiment 1 (see Fig. 1). As such, these novel exemplars were less distinct and less diagnostic of category membership.

#### Analysis

An identical analysis approach to that described in Experiment 1 was followed in Experiment 2. Experiment 2 was pre-registered prior to data analysis on the Open Science Framework at https://osf.io/9jr4c, and further details, including the analysis script, are also available at https://osf.io/kx7dc/?view_only=3da7e9a8440642dd905d25c491516095.

### Results

For the simple shape and complex shape pre-experimental sessions, overall accuracy (in the final learning block) was 82% (*SD* = 7.1) and 80% (*SD* = 6.3), respectively. To reach at least 75% accuracy for the simple and complex shapes, an average of 1.8 (*SD* = 1.4) and 2.6 (*SD* = 2.4) learning blocks was required, respectively. For the unimodal visual, auditory and bimodal category learning, overall accuracy (in the final learning block) was 86% (*SD* = 10.3), 85% (*SD* = 9.4) and 86% (*SD* = 9.9), respectively. To reach at least 75% accuracy for unimodal visual, auditory and bimodal category learning, an average of 1.5 (*SD* = 0.6), 1.8 (*SD* = 1.1) and 1.9 (*SD* = 1) learning blocks was required, respectively. In the categorisation test, overall accuracy was 86% (*SD* = 35), 67% (*SD* = 47) and 78% (*SD* = 41) for unimodal visual, auditory and bimodal categorisation, respectively. Average performance based on each participant’s ‘best’ and ‘worst’ unimodal accuracy, which was 89% (*SD* = 31) and 64% (*SD* = 48), respectively, was analysed. For approximately 83% of the sample (*n* = 58), vision was the ‘best’ modality.

**Fig. 4:**
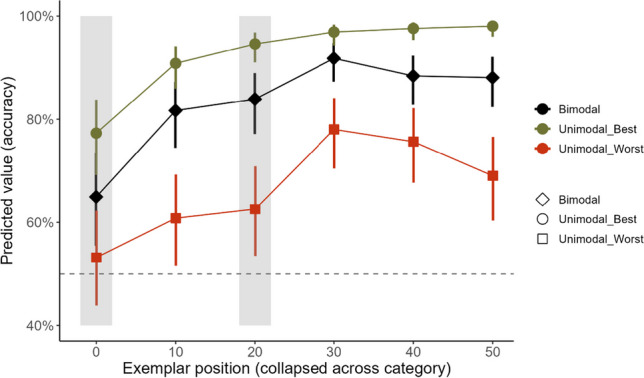
Summary of findings of mixed-effects models predicting categorisation accuracy across exemplar positions relative to the category boundary (see text) and modalities in Experiment 2. Areas shaded grey indicate the position of the novel exemplars. The dashed horizontal line demarcates chance level (50%) performance. Error bars show 95% confidence intervals

The effect of exemplar position (χ^2^(5) = 207.06, *p* < 0.001) and modality (χ^2^(2) = 72.71, *p* < 0.001; see Online Supplementary Materials) and the exemplar position*modality interaction (χ^2^(10) = 36.91, *p* < 0.001) significantly contributed to the model predicting categorisation accuracy, as shown in Fig. 4. As expected, all exemplars were more accurately categorised in the ‘best’ versus ‘worst’ unimodal condition. In addition, the exemplars at positions 50°, 40°, 30°, 20° and 10° were 3.31 (95% CI [1.38, 7.91]), 2.45 (95% CI [1.01, 5.94]), 3.16 (95% CI [1.25, 7.98]), 3.12 (95% CI [1.35, 7.22]) and 2.88 (95% CI [1.26, 6.58]) times more likely to be accurately categorised, respectively, in the bimodal compared to ‘worst’ unimodal condition. Exemplars at 50°, 40° and 20° were 85% (odds ratio = 0.15, 95% CI [0.04, 0.53]), 81% (odds ratio = 0.19, 95% CI [0.05, 0.63]) and 70% (odds ratio = 0.30, 95% CI [0.11, 0.82]) less likely to be accurately categorised, respectively, in the bimodal relative to ‘best’ unimodal condition. All other contrasts were statistically non-significant at the corrected alpha level. There was no statistically significant difference in categorisation accuracy for any modality for the novel exemplars positioned at 20° (‘best’ unimodal: odds ratio = 0.56, 95% CI [0.22, 1.46]; ‘worst’ unimodal; odds ratio = 0.93, 95% CI [0.50, 1.73]; bimodal: odds ratio = 0.86, 95% CI [0.41, 1.78]) and the most similar, previously learned exemplar positioned at 10°. Categorisation accuracy was significantly higher for the exemplar positioned at 10° compared to the novel exemplar positioned next to the category boundary at 0° in both the ‘best’ unimodal (odds ratio = 2.91, 95% CI [1.33, 6.38]) and bimodal (odds ratio = 2.40, 95% CI [1.21, 4.77]) conditions but not in the ‘worst’ unimodal condition (odds ratio = 1.36, 95% CI [0.73, 2.54].

### Discussion

As in Experiment 1, the participants were able to categorise two-dimensional exemplars based on shape, with the strongest categorisation emerging in the ‘best’ unimodal condition followed by the bimodal condition. Bimodal category learning did not benefit subsequent categorisation ability relative to the ‘best’ unimodal condition. Whether bimodal categorisation performance matched that of the ‘best’ unimodal condition again depended on the position of the exemplar. Overall, categorisation accuracy generalised well to novel exemplars, regardless of sensory modality. Although categorisation performance was less accurate for a novel exemplar positioned next to the category boundary (i.e., 0°) relative to its learned neighbour (10°) in the bimodal and best unimodal conditions, performance to exemplars positioned next to the category boundary was comparable across modalities in both experiments. This suggests a general difficulty with categorising exemplars located at the category boundary that is independent of modality.

### General discussion

This study investigated the contribution of visual, auditory and audio-visual category learning to categorisation ability of learned and novel exemplars defined by shape similarity (Li et al., 2020b). Despite evidence that multisensory cues can benefit learning (Shams & Seitz, 2008) and enhance recognition and memory abilities (Chen & Spence, 2010; Matusz et al., 2017; Meyerhoff & Huff, 2016; Molholm et al., 2004; Thelen et al., 2015), no advantage of bimodal cues was found for the categorisation of novel objects in either of the current experiments. One explanation for this is that the ‘best’ unimodal cue was sufficiently informative for shape-based categorisation alone that it ‘captured’ the performance and a bimodal advantage could not be achieved. This outcome may be predicted by reliability weighting models of sensory integration, particularly if information in one modality (shape feature or cue) is more reliable than the other (Alais et al., 2010; Helbig et al., 2012). That the ‘best’ modality was vision for the majority of both samples aligns with evidence that vision is highly informative of object shape (e.g., Klatzky et al., 1987) and that visual memories are more robust than auditory memories (Cohen et al., 2009; Meyerhoff et al., 2023; Yuval-Greenberg & Deouell, 2007, 2009). However, although degrading the visual information reduced this visual dominance (in a pilot experiment, see OSM), there was still no evidence for an overall benefit of the bimodal condition for categorisation. In Experiments 1 and 2, bimodal accuracy was either approximate to the best unimodal condition or intermediate to that of the two unimodal conditions depending on the position of the exemplar within the shape space, suggesting that the less informative sensory cue may have contributed to bimodal categorisation and that its relative weighting potentially depended on category position.

The specific mechanisms underpinning the interaction between modality and the relative position of the exemplars to the category boundary in these experiments are currently unknown. However, the present findings suggest that categorisation difficulty may be a contributory factor. For example, in Experiment 1, bimodal categorisation performance was comparable to that of the ‘best’ unimodal condition for exemplars located furthest from the boundary. Based on the structure of the circular shape space, these exemplars were most dissimilar to members of the opposite category (Li et al., 2020b). Therefore, using only the most informative sensory cue is sufficient to achieve accurate categorisation as other cues offer no further information. For example, distinguishing a tennis ball from a rugby ball is possible using visual shape information alone. However, category membership is less discernible for exemplars positioned close to the category boundary as these exemplars often share more features with exemplar members of the opposite category than their own, depending on the position within the overall shape space (Goldstone, 1994). Indeed, at the category boundary, accuracy was relatively poor and generally comparable across all modality conditions, reflecting the difficulty of categorising exemplars that are adjacent in the shape space but straddle the category division. In Experiment 1, bimodal categorisation was significantly worse than the ‘best’ unimodal categorisation for exemplar positions closer to the boundary, approximating an ‘average’ of the two unimodal conditions, suggesting that the less reliable cue was not entirely disregarded. This is consistent with the use of multiple sensory cues for category assignment as an optimal strategy to minimise category uncertainty. For example, distinguishing a tennis ball from a lacrosse ball is difficult using visual shape alone, increasing an observer’s reliance on other object features for categorisation.

However, whether the audio-visual cues were integrated or used independently to make category decisions is unclear. While a category decision could arise following the reliability-weighted integration of the sensory cues at a perceptual level, an integration of information could also manifest at a later stage, for example in memory (e.g., Meyerhoff & Huff, 2016), or via a process whereby separate criterial decisions are made for each independent sensory cue before a final selection is reached by ‘integrating’ these unimodally informed decisions (Smith et al., 2014).

Although the bimodal cues were spatially correlated, sounds do not typically represent object contours. Previous studies support the idea of correspondences between features such as pitch and shape angularity (Getz & Kubovy, 2018), but such correspondences may not be obvious in the complex stimuli used in this study. Here, pre-experiment training was included to help the participants to discern the relationship between visual and auditory shape-representations, but this training may not have been sufficiently long for integration to have occurred (Hertz & Amedi, 2015). Furthermore, the sounds may have been more temporally than spatially processed compared to vision, and these audio-visual stimuli lacked semantic meaning to further reinforce their compatibility. As such, the prior expectation that the visual and auditory cues naturally ‘belonged together’ may have been weak/absent and they may have been perceived as *independent* sources of information instead, limiting the potential for multisensory integration (e.g., in line with Bayesian models of cue integration; see Chen & Spence, 2017; Ernst & Bülthoff, 2004; Shams & Beierholm, 2010).

An intermediary performance to the bimodal condition could also indicate the division of attention across sensory modalities, producing a ‘compromise’ solution (Guest & Spence, 2003). Attention may select different features for categorisation depending on the similarity of the exemplars to members of a different category (Nosofsky, 1986). Thus, attentional processes inform the ‘weights’ assigned to stimulus dimensions during categorisation (Deng & Sloutsky, 2016), and informative category features may be more heavily ‘weighted’ in memory (Deng & Sloutsky, 2016; Folstein et al., 2015; Zmigrod & Hommel, 2013). However, cross-modal attentional splitting can also degrade recognition performance, reflecting an attentional bottleneck for information processing (Craik et al., 1996; Meyerhoff et al., 2023). It may be that, in Experiment 1, such an effect was evident for exemplars that were closer to the category boundary because they were more difficult to categorise. Nevertheless, the effect of modality was not consistent across exemplar positions in Experiment 2, suggesting that other factors may be at work. For example, which specific exemplars were previously learned may have determined the resultant category representations and the strategies employed, although the categorisation model that best fits the current data is unclear (e.g., prototype- or exemplar-based; Ashby & Maddox, 2005). Moreover, evidence for individual differences in bimodal performance in both experiments raises the possibility that different strategies were implemented (DeCaro et al., 2008; Maddox et al., 2006; Smith et al., 2014), requiring further empirical investigation.^2^ Although we encountered no issues with online testing, future studies may determine whether each individual’s task performance was, at least partially, influenced by the online nature of the study. However, given the similarity between the findings reported here and those of previous studies using cross-modal categorisation (Maddox et al., 2006; Smith et al., 2014) and perceptual tasks (e.g., Zanchi et al., 2022), we argue that our results are more likely to represent individual differences in learning strategies, the weighting of sensory cues, the distribution of attention or other cognitive factors.

Evidence for generalisation to novel category members was found even in the worst unimodal condition and regardless of which exemplars were omitted from learning, indicating flexible representations of these categories in memory. Generalisation strategies vary from drawing on simple heuristics to more in-depth analyses of object features (Morgenstern et al., 2019), but inter-object similarity plays an important role. That is, prior learning establishes the physical features common to category members and successful generalisation stems from the detection of similar features in the novel exemplars (Nosofsky, 1986; Sloutsky, 2003).

The stimuli used in this study followed a uniform distribution only, as the stimuli at the centre and extremes of the two categories were equally frequent. This sampling approach (e.g., Newell & Bülthoff, 2002) mitigates the potential influence of unequal stimulus exposure on category learning. However, exemplar distributions can influence categorisation performance, and a uniform distribution may encourage broader category generalisation and weaker category coherence than a normal distribution (Carvalho et al., 2021). This may influence the category representation itself, as increased category coherence can promote a prototype- compared to an exemplar-based representation (Bowman & Zeithamova, 2020). Further research is needed to investigate whether the level of generalisation reported here arises in response to different category distributions during audio-visual category learning, strengthening the external validity of these findings by capturing the real-world variability in perception (Carvalho et al., 2021).

It is important to note that the present findings do not preclude the possibility that bimodal cues can benefit categorical decisions in certain scenarios. For example, an advantage of multisensory cues for object memory can manifest for complex objects (Matusz et al., 2017) and for scene recognition (Meyerhoff & Huff, 2016) where semantic meaning is evident (unlike the current experiments). Thus, further empirical work is needed to better understand the mechanisms underpinning the effects observed in the present experiments, ideally with an increased number of trials and exemplars to facilitate more complex modelling of categorisation strategies in response to multisensory versus unisensory category information.

### Conclusions

This study examined whether categorisation of unfamiliar exemplars, defined by shape, is enhanced following audio-visual learning compared to visual- or auditory-only learning. No advantage of exposure to bimodal cues to category membership over the best unimodal condition was observed for categorisation accuracy. Generalisation to novel exemplars was, however, observed independent of sensory modality or exemplar position within a category. These findings indicate that additional empirical work is needed to better understand the extent to which multisensory processing influences categorisation abilities and in what contexts.

## Supplementary Information

Below is the link to the electronic supplementary material.

Supplementary file1 (DOCX 1200 kb)

## Data Availability

Materials, including soundscapes, data and analysis scripts, for both experiments are available on the Open Science Framework: https://osf.io/ujyrw/?view_only=35ca39e3c49f49558274dc0dc4bd39f9 and https://osf.io/kx7dc/?view_only=3da7e9a8440642dd905d25c491516095. Experiment 2 was preregistered on the Open Science Framework prior to data analysis, https://osf.io/9jr4c.

## Footnotes

1 No random slope for exemplar position was fitted to any model due to issues with convergence/singular fit suggesting overfitting of models to the data, in both Experiment 1 and Experiment 2.

2 In both Experiment 1 and Experiment 2, a minority of participants (*N* = 19 and *N* = 16, respectively) showed an overall benefit of bimodal cues for categorisation accuracy over the best unimodal cue, in terms of response accuracy and variance (Experiment 1: bimodal, *M* = 91%, *SD* = 29%; best unimodal, *M* = 75%, *SD* = 43%; Experiment 2: bimodal, *M* = 91%, *SD* = 29%; best unimodal, *M* = 79%, *SD* = 41%). Future studies should incorporate more trials into their paradigm to allow for modelling of whether optimal multisensory integration can occur in response to bimodal category learning (e.g., see Ernst & Bülthoff, 2004, Zanchi et al., 2022).

## Declarations

### Open Practices Statement

The data and materials for all experiments are available at https://osf.io/ujyrw/?view_only=35ca39e3c49f49558274dc0dc4bd39f9 and https://osf.io/kx7dc/?view_only=3da7e9a8440642dd905d25c491516095 and Experiment 2 was preregistered.
