# Supplementary material for: Generalisation to novel exemplars of learned shape categories based on visual and auditory spatial cues does not benefit from multisensory information
Source: Psychon Bull Rev. 2024 Aug 5;32(1):417–29. doi: 10.3758/s13423-024-02548-7 (PMC11836203; doi:10.3758/s13423-024-02548-7)
Supplement: Supplementary file 1 — Supplementary file1 (DOCX 1200 kb) [file 13423_2024_2548_MOESM1_ESM.docx]

**Supplementary Materials**

| **Table S1**  *Descriptive statistics for the final analysis sample in Experiment 1 (N = 75)* | | | | |
| --- | --- | --- | --- | --- |
| **Operating System** | ***n* (%)** | **Web browser** | ***n* (%)** |  |
| Windows | 59 (79) | Google Chrome | 62 (83) |  |
| Mac | 13 (17) | Microsoft Edge | 1 |  |
| Linux | 1 | Firefox | 10 (13) |  |
| Other/Don’t know | 2 (3) | Safari | 2 (3) |  |

| **Table S2**  *Descriptive statistics for the final analysis sample in Experiment 2 (N = 70)* | | | | |
| --- | --- | --- | --- | --- |
| **Operating system** | ***n* (%)** | **Web browser** | ***n* (%)** |  |
| Windows | 49 (70) | Google Chrome | 45 (64) |  |
| Mac | 18 (26) | Microsoft Edge | 10 (14) |  |
| Linux | 1 | Firefox | 10 (14) |  |
| Other/Don’t know | 2 (3) | Safari | 4 (6) |  |
|  |  | Other/Don’t know | 1 |  |

**Figure S1**

*Summary of findings of mixed-effects models predicting categorisation accuracy in Experiment 1*


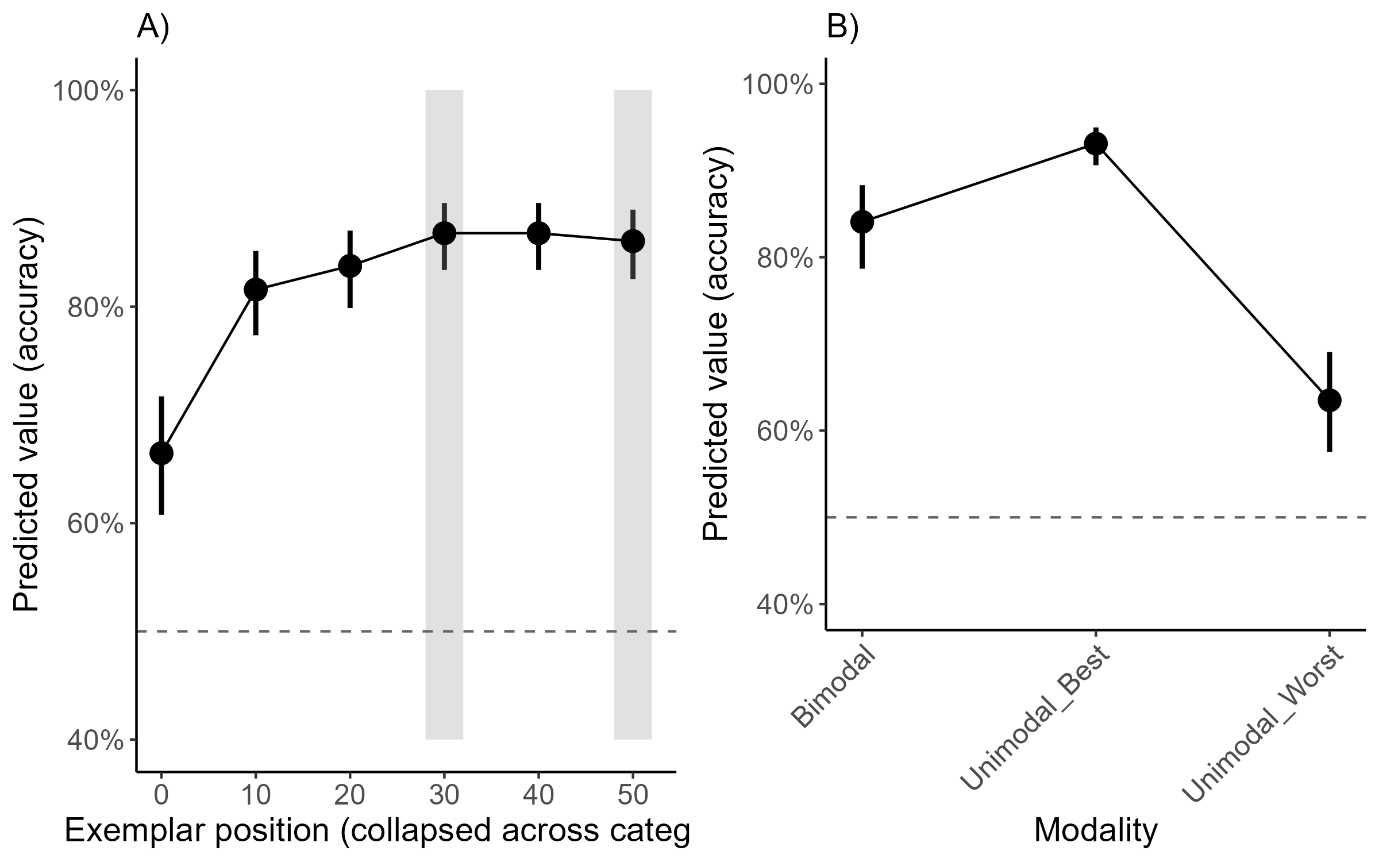


**A)** The predicted value for accuracy across exemplar position. In particular, exemplars at the category boundary (0^0^) were less likely to be accurately categorised compared to all other exemplars. Grey shaded rectangles indicate the novel exemplar positions. **B)** The predicted value for accuracy across modalities. Errors bars indicate 95% confidence intervals. The participants were 59% (odds ratio = 0.41, 95% CI [0.23,0.71]) less likely to make an accurate response in the bimodal versus best unimodal condition, 2.87 (95% CI [1.75,4.71]) times more likely to make an accurate response in the bimodal versus worst unimodal condition and 7 (95% CI [4.76,10.41]) times more likely to make an accurate response in the best versus worst unimodal condition.

**Figure S2**

*Summary of findings of mixed-effects models predicting categorisation accuracy in Experiment 2*

**
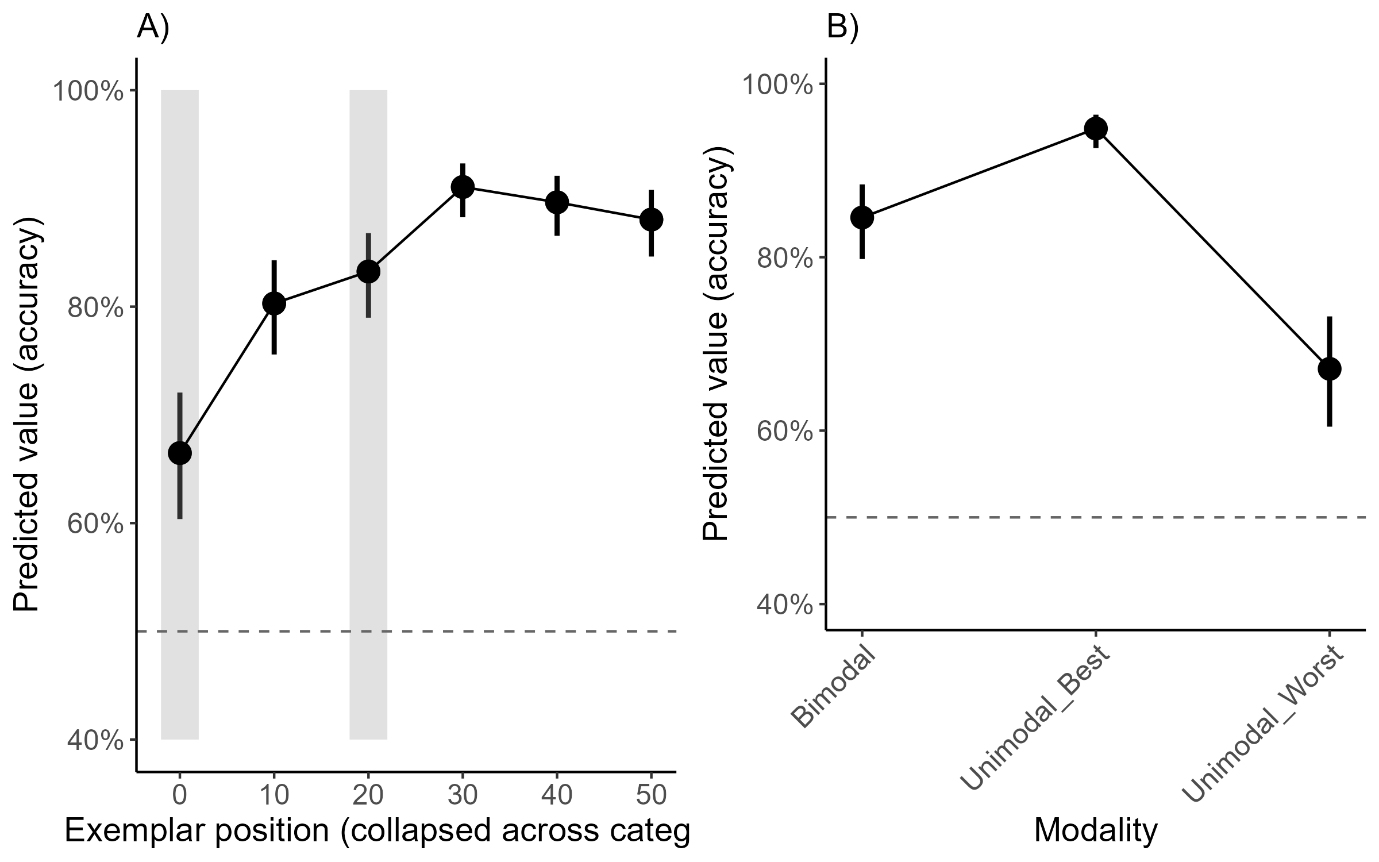
**

**A)** The predicted value for accuracy across exemplar position. In particular, the exemplars at 0^0^, 10^0^ and 20^0^ were less likely to be accurately categorised compared to other exemplars. Grey shaded rectangles indicate the novel exemplar positions. **B)** The predicted value for accuracy across modalities. Errors bars indicate 95% confidence intervals. The participants were 62% (odds ratio = 0.38, 95% CI [0.23,0.64]) less likely to make an accurate response in the bimodal versus best unimodal condition, 2.62 (95% CI [1.60,4.30]) times more likely to make an accurate response in the bimodal versus worst unimodal condition and 6.86 (95% CI [4.45,10.57]) times more likely to make an accurate response in the best versus worst unimodal condition.

| **Table S3**  *Statistically significant contrasts for the interaction between exemplar position per modality in Experiments 1 and 2 when predicting categorisation accuracy. P values are adjusted via the Bonferroni correction. All contrasts not listed were statistically non-significant at the Bonferroni corrected alpha level* | | | | | |
| --- | --- | --- | --- | --- | --- |
| **Experiment 1** |  |  |  |  |  |
| **Exemplar position** | **Modality** | **Odds ratio** | **Lower 95% CI** | **Upper 95% CI** | ***P_corr_*** |
| **50^0^** | Bimodal/worst unimodal | 3.50 | 1.48 | 8.29 | <.001 |
| **40^0^** | Bimodal/worst unimodal | 4.62 | 1.92 | 11.12 | <.001 |
| **30^0^** | Bimodal/worst unimodal | 4.23 | 1.75 | 10.20 | <.001 |
| **20^0^** | Bimodal/worst unimodal | 3.19 | 1.38 | 7.39 | .001 |
| **20^0^** | Bimodal/best unimodal | 0.26 | .09 | .74 | .002 |
| **10^0^** | Bimodal/best unimodal | 0.28 | .10 | .75 | .002 |
| **50^0^** | Best/worst unimodal | 9.11 | 3.80 | 21.81 | <.001 |
| **40^0^** | Best/worst unimodal | 9.92 | 4.11 | 23.97 | <.001 |
| **30^0^** | Best/worst unimodal | 7.15 | 3.08 | 16.62 | <.001 |
| **20^0^** | Best/worst unimodal | 12.42 | 5.11 | 30.20 | <.001 |
| **10^0^** | Best/worst unimodal | 7.62 | 3.36 | 17.30 | <.001 |
| **0^0^** | Best/worst unimodal | 3.56 | 1.79 | 7.07 | <.001 |
| **Experiment 2** |  |  |  |  |  |
| **50^0^** | Bimodal/worst unimodal | 3.31 | 1.38 | 7.91 | .001 |
| **50^0^** | Bimodal/best modality | 0.15 | .04 | .53 | <.001 |
| **40^0^** | Bimodal/worst unimodal | 2.45 | 1.01 | 5.94 | .04 |
| **40^0^** | Bimodal/best modality | 0.19 | .05 | .63 | .001 |
| **30^0^** | Bimodal/worst unimodal | 3.16 | 1.25 | 7.98 | .004 |
| **20^0^** | Bimodal/worst unimodal | 3.12 | 1.35 | 7.22 | .001 |
| **20^0^** | Bimodal/best unimodal | 0.30 | .11 | .82 | .007 |
| **10^0^** | Bimodal/worst unimodal | 2.88 | 1.26 | 6.58 | .002 |
| **50^0^** | Best/worst unimodal | 22.57 | 6.88 | 74.10 | <.001 |
| **40^0^** | Best/worst unimodal | 13.15 | 4.24 | 40.76 | <.001 |
| **30^0^** | Best/best unimodal | 8.85 | 3.05 | 25.64 | <.001 |
| **20^0^** | Best/worst unimodal | 10.52 | 4.20 | 26.37 | <.001 |
| **10^0^** | Best/worst unimodal | 6.39 | 2.78 | 14.68 | <.001 |
| **0^0^** | Best/worst unimodal | 3.00 | 1.44 | 6.24 | <.001 |

**Figure S3**

*The predicted value for the probability of a category ‘A’ response across ‘edge’ and ‘boundary’ exemplars and modalities in Experiment 1*

**
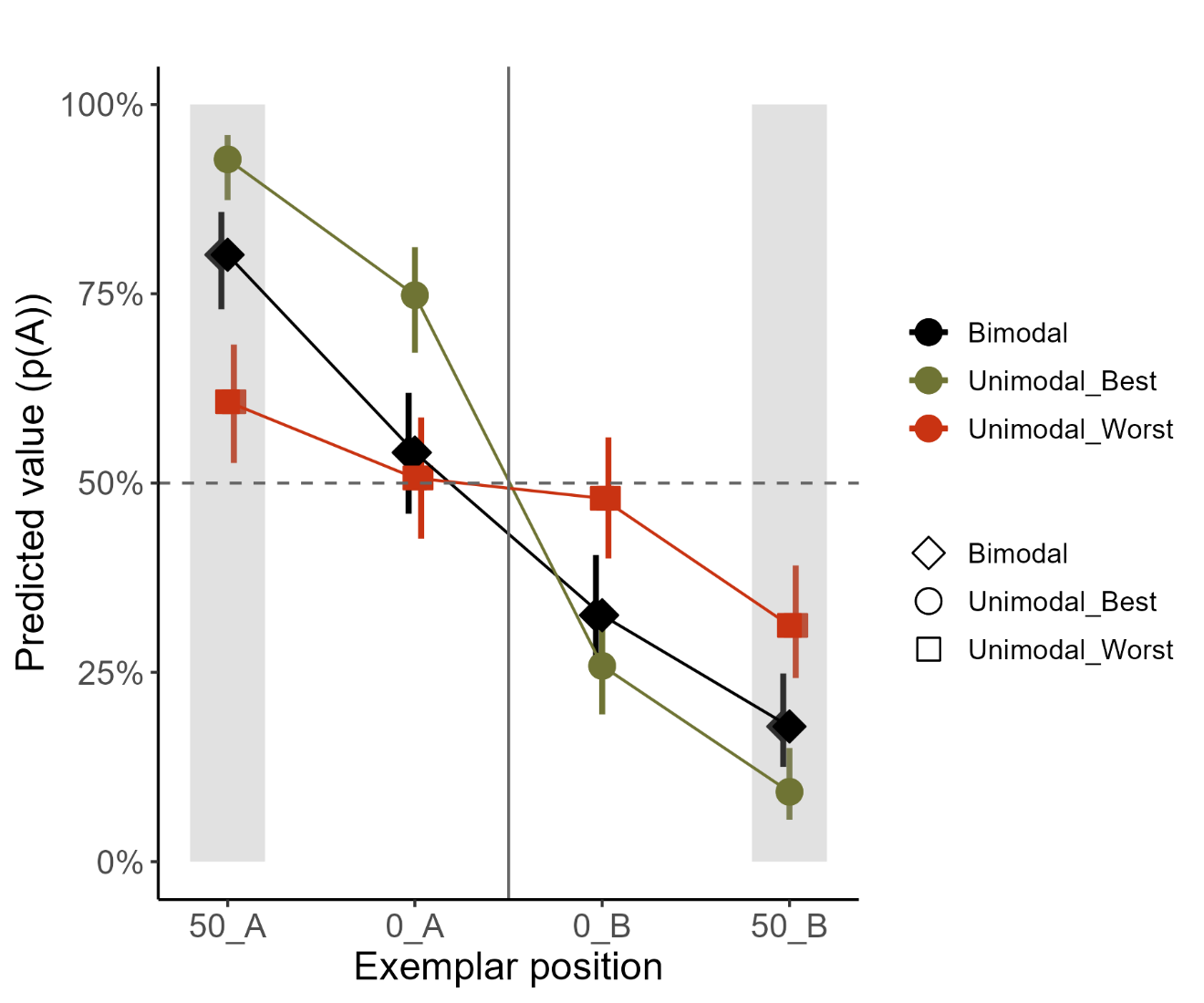
**

The solid vertical line demarcates the category boundary which was established during the learning conditions. The dashed horizontal line demarcates chance level (50%). A likelihood ratio test indicated that the exemplar position*modality interaction significantly contributed to the model predicting the probability of a category ‘A’ response (χ^2^_(6)_ = 106.38, *p* < .001). For the best unimodal condition a ‘category A’ response was 4.31 (95% CI [1.44,12.88]), 36.74 (95% CI [12.29,109.80]), and 126.05 (95% CI [35.62,446.10]) times more likely at 50^o^_A_ versus 0^o^_A_, 0^o^_B_ and 50^o^_B_ respectively, 8.52 (95% CI [3.85,18.88]) and 29.23 (95% CI [10.59,80.68]) times more likely at 0^o^_A_ versus 0^o^_B_ and 50^o^_B_ respectively and 3.43 (95% CI [1.25,9.42]) times more likely at 0^o^_B_ versus 50^o^_B_ . In the bimodal condition, a category ‘A’ response was 3.44 (95% CI [1.57,7.54]), 8.37 (95% CI [3.73,18.76]), and 18.59 (95% CI [7.66,45.12]) times more likely at 50^o^_A_ versus 0^o^_A_, 0^o^_B_ and 50^o^_B_ and 2.44 (95% CI [1.19,5]) and 5.41 (95% CI [2.42,12.11]) times more likely at 0^o^_A_ versus 0^o^_B_ and 50^o^_B_ respectively. The contrast between 0^o^_B_ and 50^o^_B_ was statistically non-significant (odds ratio = 2.22; 95% CI [.97,5.06]; p*_corr_* = .07). For the worst unimodal condition, 50^o^_A_ and 0^o^_A_were respectively 3.41 (95% CI [1.64,7.08]) and 2.26 (95% CI [1.10,4.66]) times more likely to be categorised into category ‘A’ than 50^o^_B_. All other contrasts were statistically non-significant (*p_corr_* > .05).

**Figure S4**

*The predicted value for the probability of a category ‘A’ response across ‘edge’ and ‘boundary’ exemplars and modalities in Experiment 2*

**
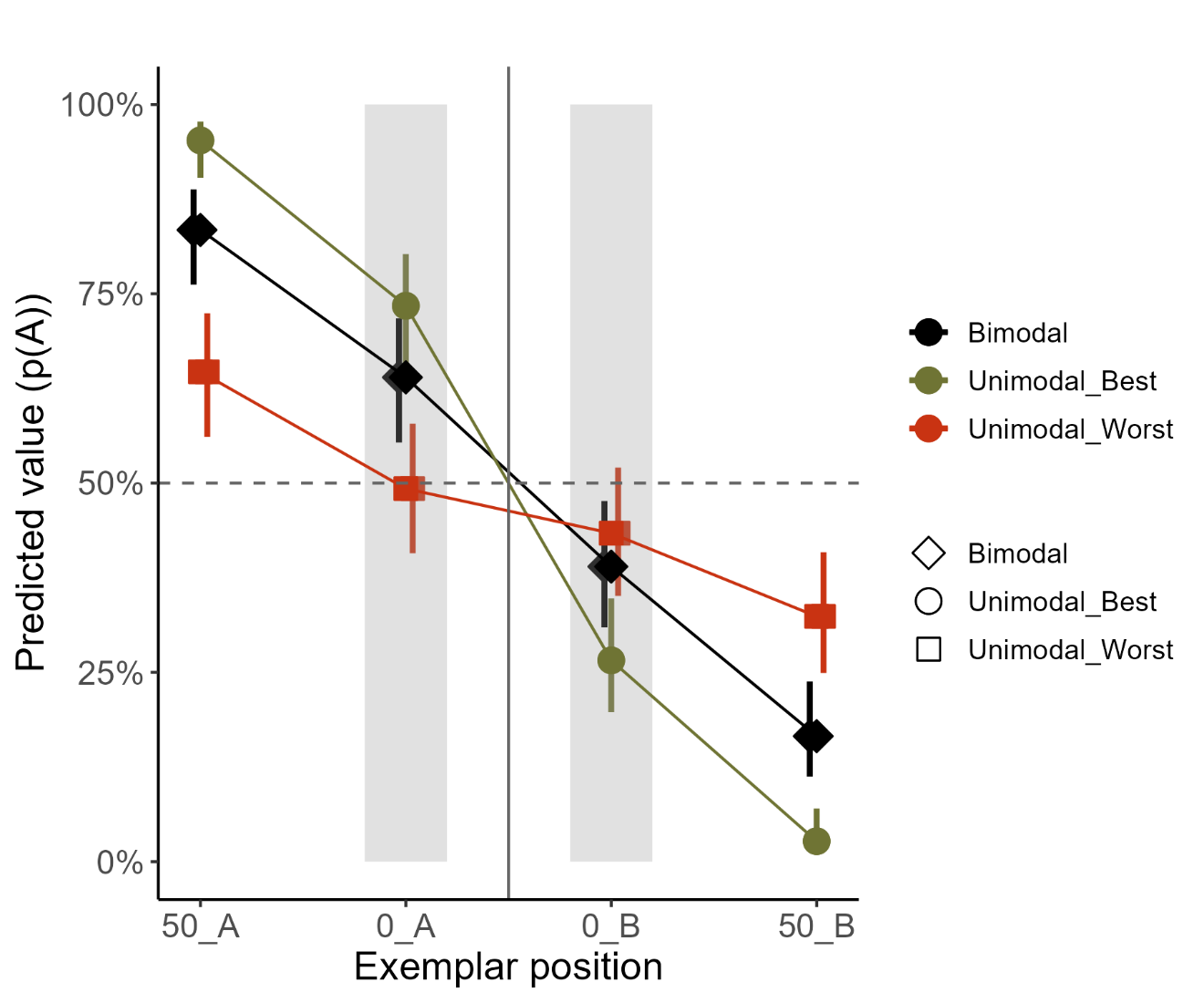
**

The solid vertical line demarcates the category boundary which was established during the learning conditions. The dashed horizontal line demarcates chance level (50%). A likelihood ratio test indicated that the exemplar position*modality interaction significantly contributed to the model predicting the probability of a category ‘A’ response (χ^2^_(6)_ = 120.97, *p* < .001). In the best unimodal condition, a category ‘A’ response was 7.27 (95% CI [1.99,26.66]), 55.48 (95% CI [15.07,204.29]), and 724.12 (95% CI [106.04,4944.76]) times more likely at 50^o^_A_ versus 0^o^_A_,  0^o^_B_ and 50^o^_B_ respectively , 7.63 (95% CI [3.37,17.28]) and 99.54 (95% CI [19.50,508.04]) times more likely at 0^o^_A_ versus 0^o^_B_ and 50^o^_B_ respectively and 13.05 (95% CI [2.57,66.34]) times more likely at 0^o^_B_ versus 50^o^_B_ respectively. In the bimodal condition, a category ‘A’ response was 2.84 (95% CI [1.20,6.72]), 7.89 (95% CI [3.34,18.64]), and 25.35 (95% CI [9.67,66.46]) times more likely at 50^o^_A_ versus 0^o^_A_,  0^o^_B_ and 50^o^_B_ respectively, 2.78 (95% CI [1.31,5.88]) and 8.94 (95% CI [3.76,21.22]) times more likely at 0^o^_A_ versus 0^o^_B_ and 50^o^_B_ respectively and 3.21 (95% CI [1.36,7.58]) times more likely at 0^o^_B_ versus 50^o^_B_. In the worst unimodal condition, a category ‘A’ response was 2.39 (95% CI [1.14,5.04]) and 3.83 (95% CI [1.78,8.23]) times more likely at 50^o^_A_ versus 0^o^_B_ and 50^o^_B_ respectively. All other contrasts were statistically non-significant (*p_corr_* > .05).

**Exploratory analyses – Experiment 1**

We conducted an exploratory analysis to investigate whether categorisation reaction times significantly differed across modalities and exemplar positions. Linear-mixed effects regression models with the fixed effect of modality (bimodal, best unimodal, worst unimodal) and exemplar position, adjusted for age (scaled), participant sex and category and including a random intercept across participants and a random slope for modality was fitted using restricted maximum likelihood estimation. Model comparisons were conducted with maximum likelihood estimation fits. A likelihood ratio test indicated that the exemplar position*modality interaction did not significantly contribute to the model predicting categorisation reaction times (χ^2^_(10)_ = 6.00, *p* = .82). However, likelihood ratio tests indicated that exemplar position (χ^2^_(5)_ = 16.38, *p* = .006) and modality (χ^2^_(2)_ = 11.82, *p* = .003) both predicted reaction times. The participants were faster categorising the exemplars at ‘50^0^’ (β = -.097, 95% CI[-.190,-.004], *p_corr_* = .03) and ‘40^0^’ (β = -.102, 95% CI[-.195,-.010], *p_corr_* = .02) respectively compared to 0^0^, slower categorising in the bimodal versus best unimodal conditions (β = .264, 95% CI[.026, .502], *p_corr_* = .025) and faster categorising in the best versus worst unimodal conditions (β = -.489, 95% CI [-.833,-.146], *p_corr_* = .002).

We also conducted an exploratory analysis to investigate whether the performance variability, based on standard deviation, significantly differed across modalities^[[1]](#footnote-1)^. Linear-mixed effects regression models with the fixed effect of modality (bimodal, best unimodal, worst unimodal), adjusted for age (scaled), participant sex and category and including a random intercept across participants was fitted using restricted maximum likelihood estimation. Model comparisons were conducted with maximum likelihood estimation fits. A likelihood ratio test indicated that the effect of modality significantly contributed to the model predicting categorisation variability (χ^2^_(2)_ = 85.25, *p* < .001). The participants were overall more variable categorising in the bimodal versus best unimodal condition (β = .08, 95% CI[.04, .13], *p_corr_* < .001), less variable categorising in the bimodal versus worst unimodal condition (β = -.11, 95% CI [-.16,-.06], *p_corr_* < .001) and less variable categorising in the best versus worst unimodal condition (β = -.19, 95% CI [-.24,-.15], *p_corr_* < .001).

**Exploratory analyses – Experiment 2**

A likelihood ratio test indicated that the exemplar position*modality interaction did not significantly contribute to the model predicting categorisation reaction times (χ^2^_(10)_ = 9.33, *p* = .50). Subsequent likelihood ratio tests indicated that exemplar position did not predict reaction times (χ^2^_(5)_ = 6.33, *p* = .28) although modality did (χ^2^_(2)_ = 12.59, *p* = .002). The participants were slower in the bimodal versus best unimodal condition (β = .318, 95% CI[.028, .608], *p_corr_ =* .03) and faster in the best relative to worst unimodal condition (β = -.553, 95% CI [-.935,-.171], *p_corr_ =* .002).

As in Experiment 1, we conducted an exploratory analysis to investigate whether the performance variability, based on standard deviation, significantly differed across modalities. Linear-mixed effects regression models with the fixed effect of modality (bimodal, best unimodal, worst unimodal), adjusted for age (scaled), participant sex and category and including a random effect of intercept across participants was fitted using restricted maximum likelihood estimation. Model comparisons were conducted with maximum likelihood estimation fits. A likelihood ratio test indicated that the effect of modality significantly contributed to the model predicting categorisation variability (χ^2^_(2)_ = 55.63, *p* < .001). The participants were overall more variable categorising in the bimodal versus best unimodal condition (β = .09, 95% CI[.04, .15], *p_corr_* < .001), less variable categorising in the bimodal versus worst unimodal condition (β = -.08, 95% CI [-.13,-.03], *p_corr_* = .001) and less variable categorising in the best versus worst unimodal condition (β = -.17, 95% CI [-.23,-.12], *p_corr_* < .001).

**Experiment 3 (exploratory)**

**Methods**

**Participants**

In total, 32 individuals partook in the experiment, all of whom were recruited on Prolific (https://www.prolific.co/). Inclusion criteria were identical to those of Experiments 1 and 2. Participants who partook in Experiment 1 or Experiment 2 were excluded from partaking in Experiment 3. Data were omitted from 8 participants who did not complete the experiment. The final analysis sample consisted of 24 participants (mean age = 32.58, years, *SD* = 11.82; 50% female). All participants provided informed consent before partaking in the experiment and were fully debriefed once the experiment was completed.

| **Table S4**  *Descriptive statistics for the final analysis sample in Experiment 3 (N = 24)* | | | | |
| --- | --- | --- | --- | --- |
| **Age (mean, SD)** | **Sex** | ***n* (%)** | **Education** | ***n* (%)** |
| 32.58 (11.82) | Male | 12 (50) | Primary/none | 2 (8) |
|  | Female | 12 (50) | Secondary | 22 (92) |
|  |  |  | Tertiary | / |
| **Vision problem** | ***n* (%)** | **Hearing problem** | ***n* (%)** |  |
| Yes | 4 (17) | Yes | / |  |
| No | 20 (83) | No | 24 (100) |  |
| **Operating system** | ***n* (%)** | **Web browser** | ***n* (%)** |  |
| Windows | 17 (71) | Google Chrome | 18 (75) |  |
| Mac | 7 (29) | Microsoft Edge | 4 (17) |  |
| Linux | / | Firefox | 2 (8) |  |
| Other/Don’t know | / | Safari | / |  |
|  |  | Opera | / |  |
|  |  | Other/Don’t know | / |  |
| **No. bimodal learning blocks (mean, SD)** | **No. audition learning blocks (mean, SD)** | **No. vision learning blocks (mean, SD)** | **Bimodal accuracy (mean, SD)** | **Auditory accuracy** **(mean, SD)** |
| 2.38 (1.58) | 3.21 (2.06) | 2.42 (1.32) | 77% (42) | 76% (43) |
| **Vision accuracy** | **Best modality accuracy** | **Worst modality accuracy** |  |  |
| 75% (44) | 85% (36) | 66% (48) |  |  |
|  | | | | |

**Stimuli**

The same exemplars described in Experiments 1 and 2 were used in Experiment 3. However, the visual exemplars were now overlayed with visual noise to reduce the reliability of the visual shape-cues for categorisation. Visual noise was added using Gimp (version 2.10.32) and the Hurl noise filter (repeat = 4, randomization percentage = 42 and subsequently desaturated) which randomizes a fraction of pixels in the image. In an online pilot experiment (*N* = 12), the application of this filter reduced participants’ ability to correctly discriminate whether two shapes, located 10 degrees apart in the visual shape space, were the same or different to each other by approximately 9% (from 75% accuracy with no filter to 66% accuracy with a full filter; *p* = .002). Importantly, this filter significantly reduced visual shape-based discrimination performance without completely rendering the shapes imperceptible (as participants still performed > 50%).

**Procedure**

The same procedure described in Experiments 1 and 2 was followed in Experiment 3, featuring a within-subjects design. Once again, the main experiment consisted of category learning and a subsequent categorisation test. However, as the participants demonstrated an ability to adequately generalise to novel exemplars in both prior experiments, we dropped this manipulation from Experiment 3. As such, all shapes were learned and subsequently tested.

**Figure S5**

*Examples of masked visual shapes from Experiment 3*


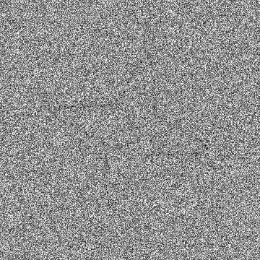

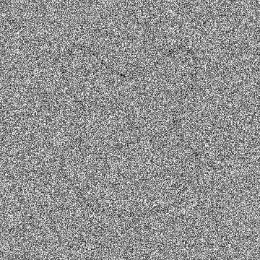

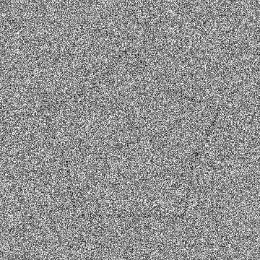


**Results**

Overall categorisation accuracy was 75% (*SD* = 44%), 76% (*SD* = 43%) and 77% (*SD* = 42%) for the visual, auditory and bimodal conditions. However, there was still an overall bias towards one modality (best modality: *M* = 85%, *SD* = 36%; worst modality: *M* = 66%, *SD* = 48%), with the best modality evenly split between vision and audition, and no clear advantage of the bimodal condition.

**A note on intermediary bimodal performance**

A cue-switching strategy (Ernst & Bülthoff, 2004), whereby participants switch between sensory modalities on a trial by trial basis based on cue reliability, or optimal multisensory integration, referring to the linear fusion of sensory cues based on their reliability, can also give rise to a performance average in a bimodal condition (Ernst & Bülthoff, 2004) but these explanations are unlikely to explain our findings. Firstly, it seems unlikely that participants would switch exclusively to using the worst cue when categorising stimuli close to the category boundary. Secondly, optimal integration should arise in response to sensory cues that are approximated in reliability and should also reduce response variability (Ernst & Bülthoff, 2004). In our experiments, there was always a significant difference in performance between the unimodal conditions and we observed no reduction in overall variance for bimodal categorisation. Furthermore, if the sensory cues were perceived as independent sources of category information, this would preclude optimal multisensory integration (Ernst & Bülthoff, 2004). We also argue against a general effect of cross-modal distraction which would be expected to manifest comparably across exemplars and experiments.

**Pre-experimental session stimuli**

**
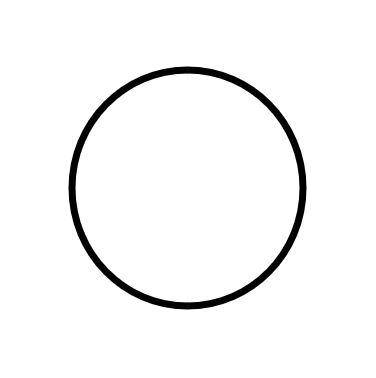

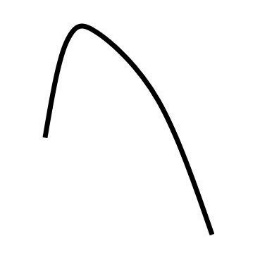

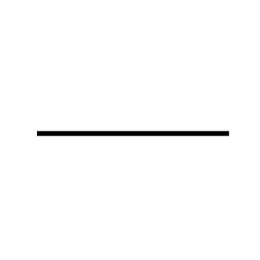

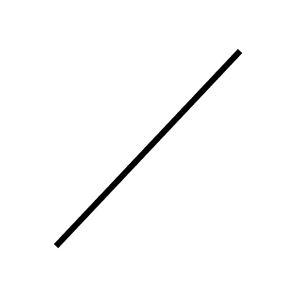

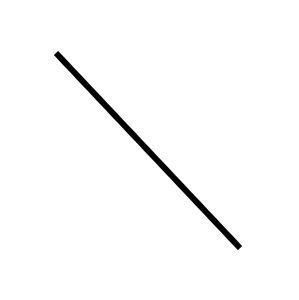

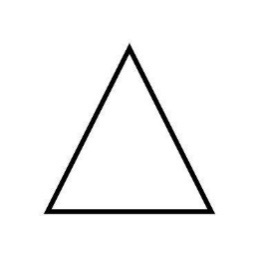

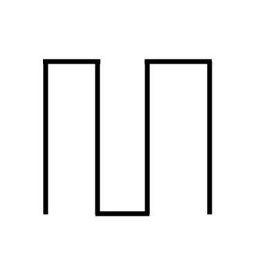

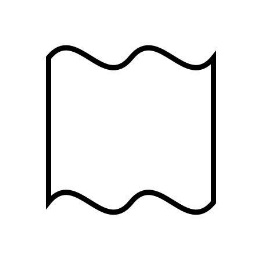
**

**
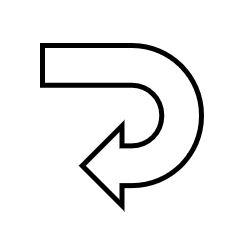

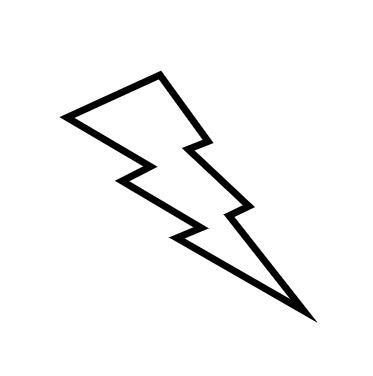

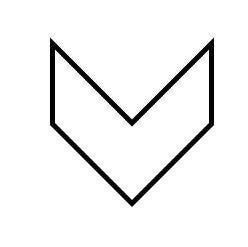

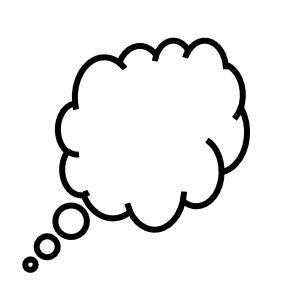

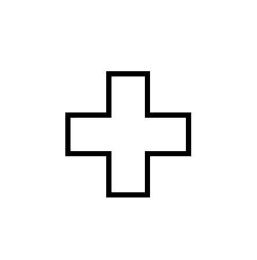

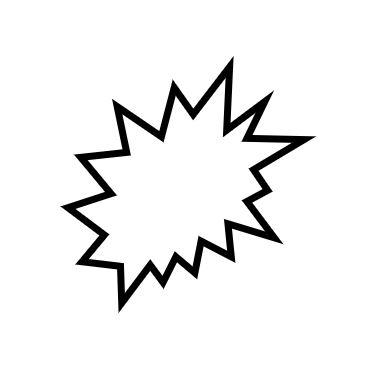

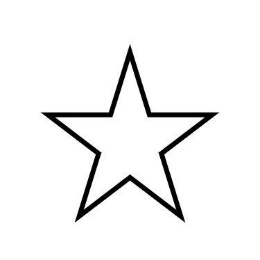

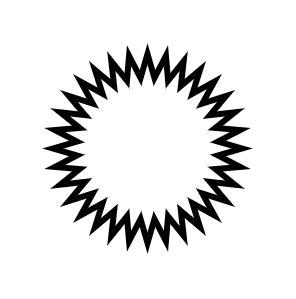
**

1. We could not investigate variance on an exemplar position*modality basis given that there were only 2 trials for each stimulus per modality condition. [↑](#footnote-ref-1)
